# The Herbarium 2021 Half–Earth Challenge Dataset and Machine Learning Competition

**DOI:** 10.3389/fpls.2021.787127

**Published:** 2022-02-01

**Authors:** Riccardo de Lutio, John Y. Park, Kimberly A. Watson, Stefano D'Aronco, Jan D. Wegner, Jan J. Wieringa, Melissa Tulig, Richard L. Pyle, Timothy J. Gallaher, Gillian Brown, Gordon Guymer, Andrew Franks, Dhahara Ranatunga, Yumiko Baba, Serge J. Belongie, Fabián A. Michelangeli, Barbara A. Ambrose, Damon P. Little

**Affiliations:** ^1^EcoVision Lab, Department of Civil, Environmental and Geomatic Engineering, ETH Zürich, Zurich, Switzerland; ^2^New York Botanical Garden, Bronx, NY, United States; ^3^Faculty of Science, Institute for Computational Science, University of Zurich, Zurich, Switzerland; ^4^Naturalis Biodiversity Center, Leiden, Netherlands; ^5^Bishop Museum, Honolulu, HI, United States; ^6^Queensland Herbarium, Department of Environment and Science, Toowong, QLD, Australia; ^7^Auckland War Memorial Museum Tāmaki Paenga Hira, Auckland, New Zealand; ^8^Department of Computer Science, University of Copenhagen, and Pioneer Centre for AI, Copenhagen, Denmark

**Keywords:** herbarium specimen image, fine-grained visual categorization, machine learning competition, hierarchical classification, datasets

## Abstract

Herbarium sheets present a unique view of the world's botanical history, evolution, and biodiversity. This makes them an all–important data source for botanical research. With the increased digitization of herbaria worldwide and advances in the domain of fine–grained visual classification which can facilitate automatic identification of herbarium specimen images, there are many opportunities for supporting and expanding research in this field. However, existing datasets are either too small, or not diverse enough, in terms of represented taxa, geographic distribution, and imaging protocols. Furthermore, aggregating datasets is difficult as taxa are recognized under a multitude of names and must be aligned to a common reference. We introduce the Herbarium 2021 Half–Earth dataset: the largest and most diverse dataset of herbarium specimen images, to date, for automatic taxon recognition. We also present the results of the Herbarium 2021 Half–Earth challenge, a competition that was part of the Eighth Workshop on Fine-Grained Visual Categorization (FGVC8) and hosted by Kaggle to encourage the development of models to automatically identify taxa from herbarium sheet images.

## 1. Introduction

Herbaria, like other natural history collections, are immense primary data repositories documenting biodiversity across space and time over the last 500 years (Stefanaki et al., 2019). Each specimen contains a wealth of information including geographic occurrence data, phenotype, genotype, phenological status, and biotic interactions (Funk, 2003; Heberling and Burke, 2019). Collectively herbarium specimens are analyzed for studies in taxonomy, systematics, floristics, ecology, phenology, conservation, and global environmental change (Funk, 2003; Calinger et al., 2013; Willis et al., 2017; Lang et al., 2019; Albani Rocchetti et al., 2021).

Worldwide efforts to digitize and electronically mobilize biodiversity data for the estimated 396 million herbarium specimens, housed in 3,400 herbaria (Thiers, 2021), have greatly amplified their use in research (Heberling et al., 2019; Nelson and Ellis, 2019), including projects to understand, predict, and ameliorate increasing environmental threats to biodiversity (Intergovernmental Science–Policy Platform on Biodiversity and Ecosystem Services, 2019; Lang et al., 2019). Plants are essential to life on Earth, yet an estimated 37–44% of all vascular plant species are threatened with extinction (Nic Lughadha et al., 2020), underscoring the urgency to identify and classify the estimated 70,000 flowering plant species not yet described (Bebber et al., 2010; Joppa et al., 2011). Half of these new species are predicted to be already preserved in herbaria, awaiting an average of 35 years for detection and description from the date of first specimen collection (Bebber et al., 2010). Contributing to this delay is the dwindling number of taxonomists with broad plant identification skills to recognize new species, who are under ever increasing demands on their time and expertise (Secretariat of the Convention on Biological Diversity, 2007).

Recent advances in machine learning and computer vision as well as increased biodiversity data mobilization through global data aggregators, such as the Global Biodiversity Information Facility (GBIF), enable the development of models to address a variety of plant–science–related questions and potentially overcome such “taxonomic impediments” (Secretariat of the Convention on Biological Diversity, 2007; Heberling et al., 2021). For example, the automatic identification of specimens has shown particularly promising results from learning–based approaches (review by Wäldchen and Mäder, 2018). Many studies have focused on small sets of closely–related plant taxa (Clark et al., 2012; Nasir et al., 2014; Unger et al., 2016; Kho et al., 2017; Schuettpelz et al., 2017; Pryer et al., 2020) whereas others tackle the more challenging problem of automatic identification of a large number of taxa (Carranza-Rojas et al., 2017; Younis et al., 2018; Little et al., 2020). Many automatic identification studies focus on recognition from leaves alone (Wijesingha and Marikar, 2012; Nasir et al., 2014; Unger et al., 2016; Wilf et al., 2016; Kho et al., 2017). Similar techniques have also been used for phenological studies and trait recognition (Clark et al., 2012; Ubbens and Stavness, 2017; Younis et al., 2018; Lorieul et al., 2019; Brenskelle et al., 2020; Davis et al., 2020; Goëau et al., 2020; Pearson et al., 2020; Pryer et al., 2020).

Citizen science initiatives, such as iNaturalist (Horn et al., 2018), Pl@ntNet (Joly et al., 2016), and ObsIdentify (Hogeweg et al., 2019), have popularized species recognition as a challenging real–world classification task among the computer vision community. They are particularly popular because of the size as well as the imbalanced and fine–grained nature of their respective datasets. Through a series of online algorithm competitions (e.g., Horn et al., 2018; Little et al., 2020), automated identification techniques have become increasingly accurate.

Existing digitized herbarium specimen datasets designed for computer vision approaches present some limitations: they are either small, targeted at specific taxa, representative of only a small geographic region, or contain images produced using only one imaging protocol (generally institution specific; Table 1). In this paper we introduce the Herbarium 2021 Half–Earth dataset, which aims to address the limitations aforementioned and is the largest and most diverse dataset of herbarium specimen images for automatic taxon recognition to date. We also present the results from the challenge of the same name: the Herbarium 2021 Half–Earth challenge, a competition that was organized as part of the 8*th* workshop for Fine–Grained Visual Categorization at the Computer Vision and Pattern Recognition conference (CVPR) in 2021. The competition was hosted on Kaggle^1^ and took place between March 10*th* and May 27*th* 2021.

**Table 1:** Summary of existing herbarium sheet image datasets.

**Dataset**	**Images**	**Taxa**	**Vascular plant representation (%)**	**Institutions**	**Geographic range**
Dillen et al., 2019	1,900	1,580	0.34	9	Global
Herbarium 255 (Carranza-Rojas et al., 2017)	11,071	255	0.05	1	Costa Rica
Herbarium 1K (Carranza-Rojas et al., 2017)	253,733	1,204	0.26	1	France
Herbarium 2019 (Tan et al., 2019)	46,000	680	0.15	1	Americas
Herbarium 2020	1,169,039	32,094	6.85	1	Americas
Herbarium 2021	2,500,779	64,500	13.76	5	Americas, Oceania, and Pacific

*The percent of vascular plant taxa represented is based on the 468,759 LCVP (Freiberg et al., 2020) “accepted” and “unresolved” taxa. Because different taxonomies were used as standards for the various datasets, the reported percentage can only be considered an approximation. Note that the Herbarium 2019 dataset focuses on the flowering plant family Melastomataceae, while the other datasets include representatives across vascular plants*.

The goal of the competition was to encourage the development of models to automatically identify a very large number of taxa from herbarium sheet images, and evaluate which deep learning approaches have the best performance in this setting. This is the third iteration of the Herbarium challenge: the Herbarium 2019 challenge (Tan et al., 2019; Little et al., 2020) focused on the flowering plant family Melastomataceae and contained 46,469 digitally imaged herbarium specimens representing 683 species. The Melastomataceae is a large family with 166 recognized genera and 5,892 species (Freiberg et al., 2020). The Herbarium 2020 dataset contained 1,169,039 images representing 32,094 plant species. This challenge focused on vascular land plants of the Americas. Compared to the previous datasets the 2021 Half–Earth dataset is larger in terms of both number of taxa, and number of images, with 2,500,779 images and 64,500 taxa. After introducing the dataset and presenting the results of the competition, we discuss possible outlooks in order to leverage the full potential of deep learning models and herbarium data.

## 2. Methods

### 2.1. The Herbarium 2021 Half–Earth Dataset

**Figure 1:**
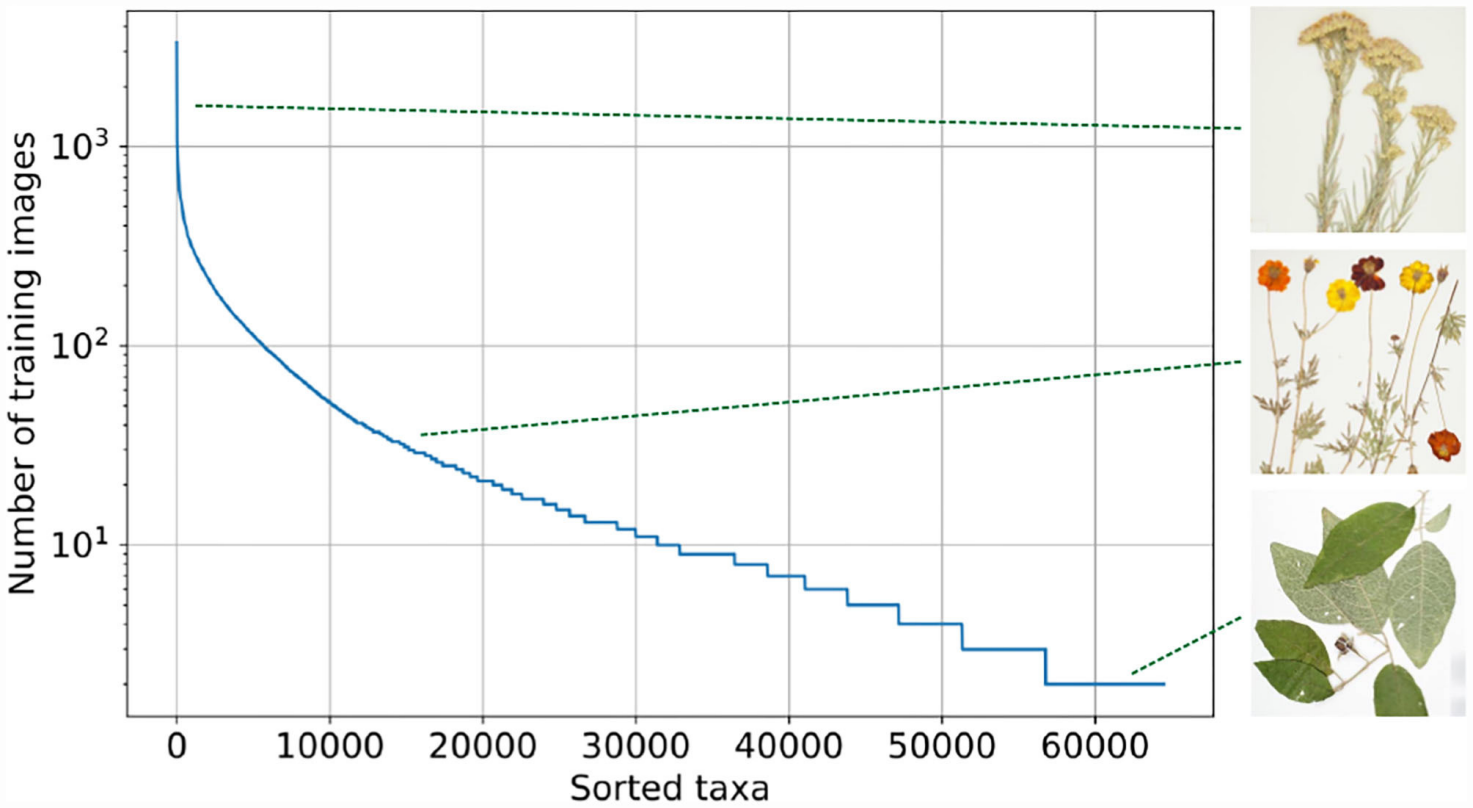
Distribution of training images per taxon. The Herbarium 2021 Half–Earth dataset is highly imbalanced. Featured taxa are from top to bottom: *Ericameria nauseosa* (Pall. ex Pursh) G.L. Nesom & G.I. Baird (Asteraceae), *Bidens sulphurea* (Cav.) Sch. Bip. (Asteraceae), and *Solanum rixosum* A.R. Bean (Solanaceae). Taxon names are usually followed by name of person(s) first formally describing the taxon in the scientific literature. Here, higher level hierarchy of each taxon is followed by family name in parentheses.

**Figure 2:**
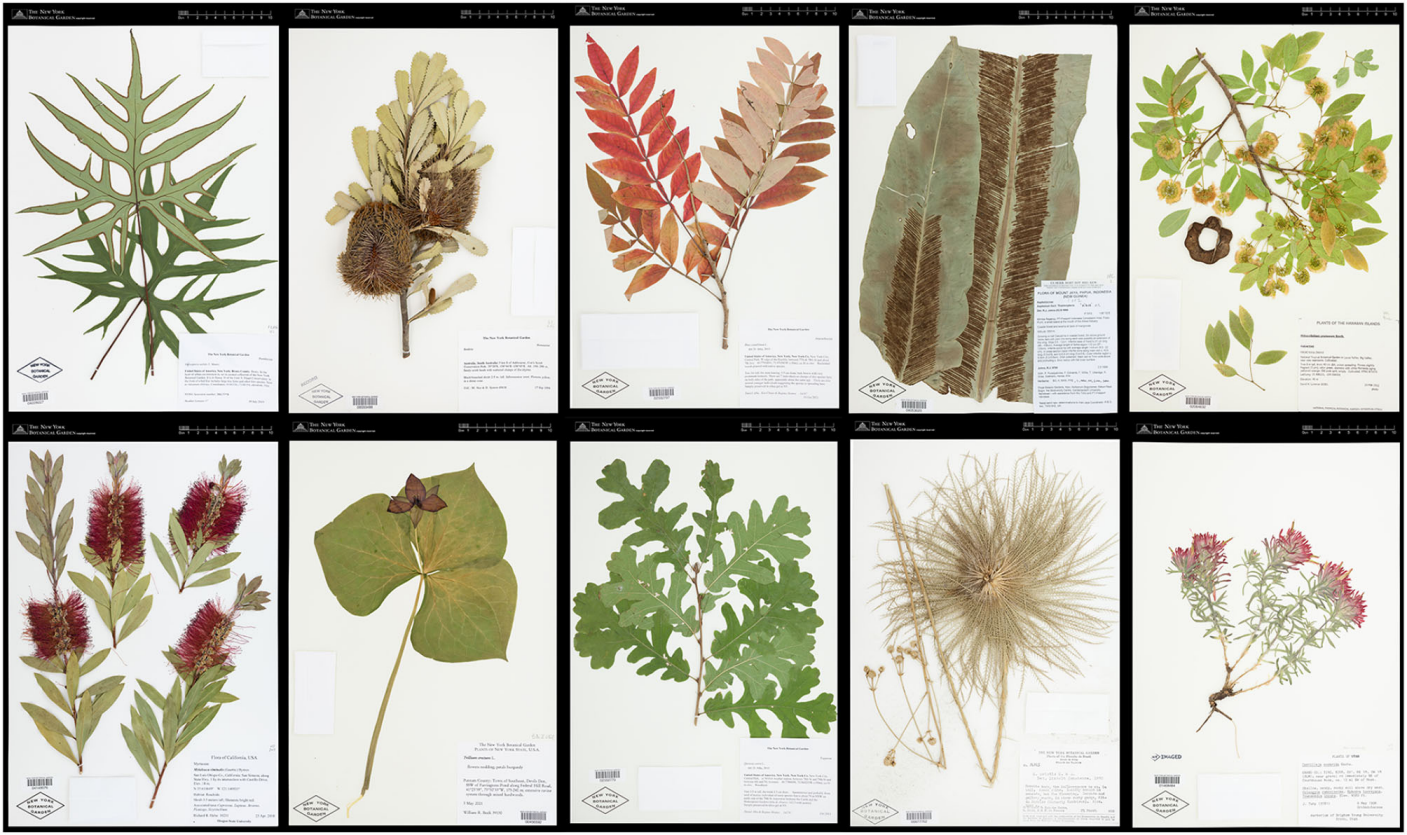
Example of images in the Herbarium 2021 Half–Earth dataset.

The Herbarium 2021 Half–Earth dataset^2^ includes more than 2.5 million images of vascular plant specimens (including lycophytes, ferns, gymnosperms, and flowering plants) representing 64,500 taxa from the Americas, Oceania, and Pacific^3^. The images are provided by the New York Botanical Garden (NY), Bishop Museum (BPBM), Naturalis Biodiversity Center (NL), Queensland Herbarium (BRI), and Auckland War Memorial Museum (AK). The most exact labels are, in many cases, infraspecific (subspecies, varieties, forms, etc.) or nothospecies (hybrids), neither of which can be characterized as “species”, thus the terms “taxon” and “taxa” are used as generic descriptors of taxonomic labels. In addition to labels for species–level and below, labels at higher levels in the taxonomic hierarchy are also included: family and order. This allows for experimentation with methods that address label hierarchy and label similarity. These labels may also be supplemented by more fine–grained estimates of difference among taxa available from other sources (e.g., Jin and Qian, 2019). The dataset is characterized by a skewed long tail distribution (Figure 1). Whereas some taxa can be represented by more than 1,000 images, other taxa have only three images. This dataset includes only images of vascular plants—the group of plants that includes lycophytes, ferns, gymnosperms, and flowering plants (Figure 2).

#### 2.1.1. Dataset Challenges

**Figure 3:**
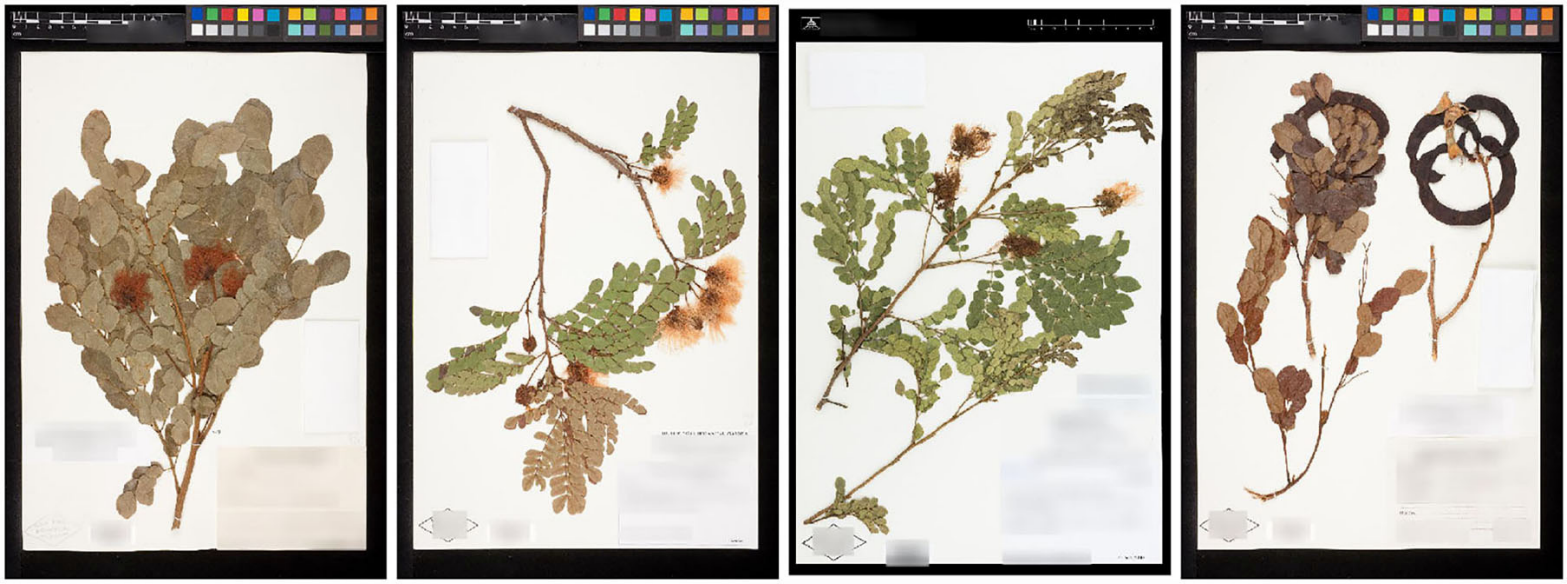
Example of visually different images corresponding to the same species: *Abarema brachystachya* (DC.) Barneby and J. W. Grimes (Fabaceae). The observed differences are primarily due to different reproductive stages: early flowering, late flowering, and fruit.

**Figure 4:**
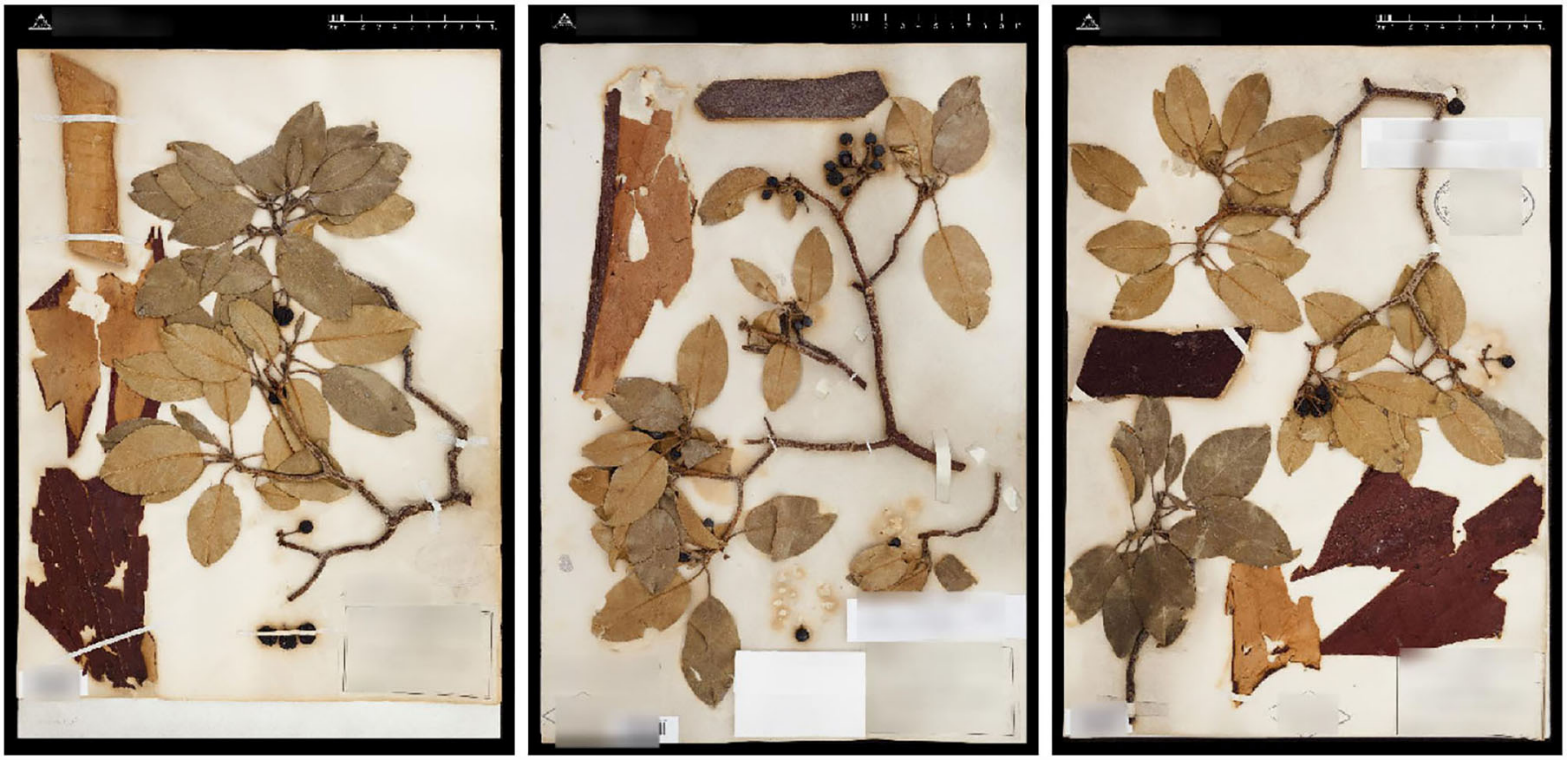
Different specimens of *Arbutus xalapensis* Kunth (Ericaceae) made from the same individual plant at the same time by the same collector using the same pressing, drying, and mounting protocol.

**Figure 5:**
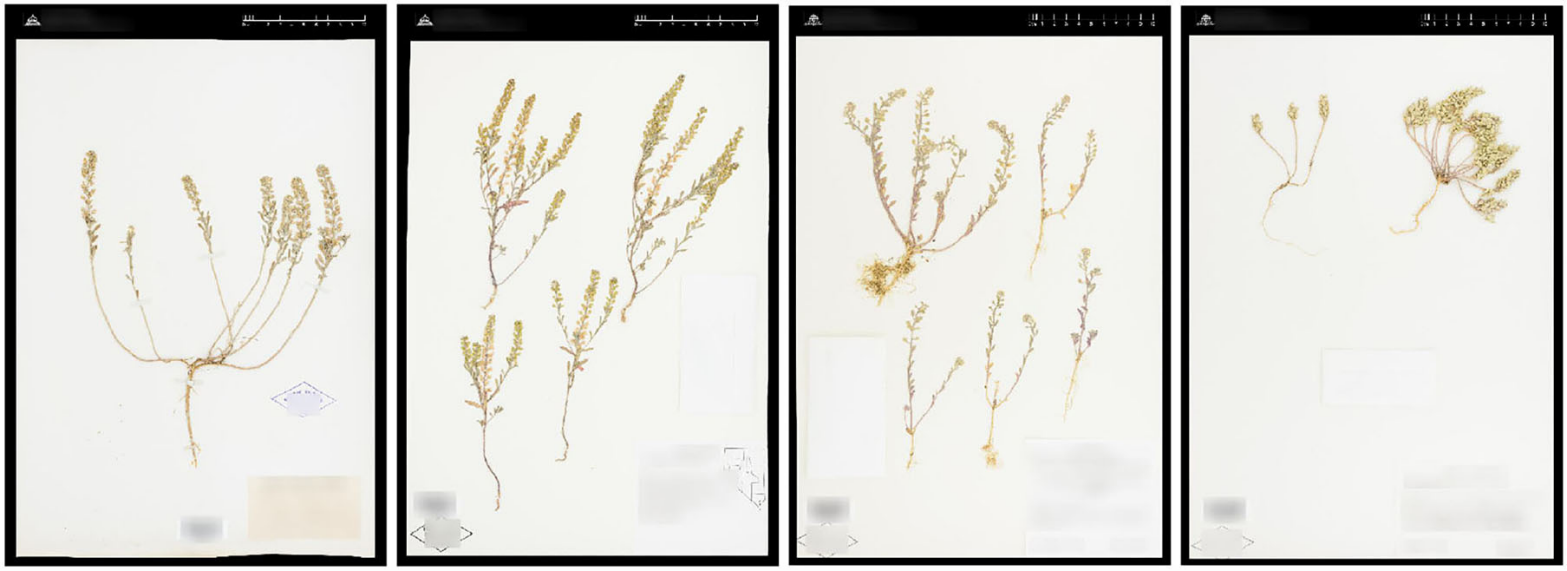
Example of visually similar images from different *Alyssum* species (Brassicaceae): *A. alyssoides* (L.) L., *A. desertorum* Stapf, *A. simplex* Rudolphi, *A. szovitsianum* Fisch. and C. A. Mey.

The Herbarium 2021 Half–Earth dataset is challenging for multiple reasons. First, of course, the large imbalance (Figure 1): the imbalance factor (ratio of the number of images for the most represented class to the number of images for the least represented class) for the dataset is 1,654.5. Second, the variation within species is high: herbarium specimens capture plants at different growth–stages (e.g., juvenile vs. adult), with different sets of plant parts (e.g., leaves and flowers vs. leaves and fruit; Figure 3) or simply different individuals can present different visual appearances. In addition, the techniques used to press, dry, and mount specimens vary among collectors and collecting expeditions—these differences can change the appearance of specimens dramatically (e.g., collecting in alcohol often causes leaves to turn black). Arbitrary aesthetic decisions made while processing specimens can result in specimens that differ dramatically in appearance even though they are simply different parts of the same individual plant (Figure 4). In a herbarium collection, every attempt to conserve dried specimens is made, but in practice older specimens become more fragile and suffer damage as they age leading to some specimens being less complete and more damaged than others. Third, the visual similarity among species can be high (Figure 5). Finally, the diagnostic morphological features that botanists use to identify species are often very small and thus require a model that is able to handle high–resolution images and can focus on specific details (Cope et al., 2012; Wäldchen and Mäder, 2018).

#### 2.1.2. Data Preprocessing

**Figure 6:**
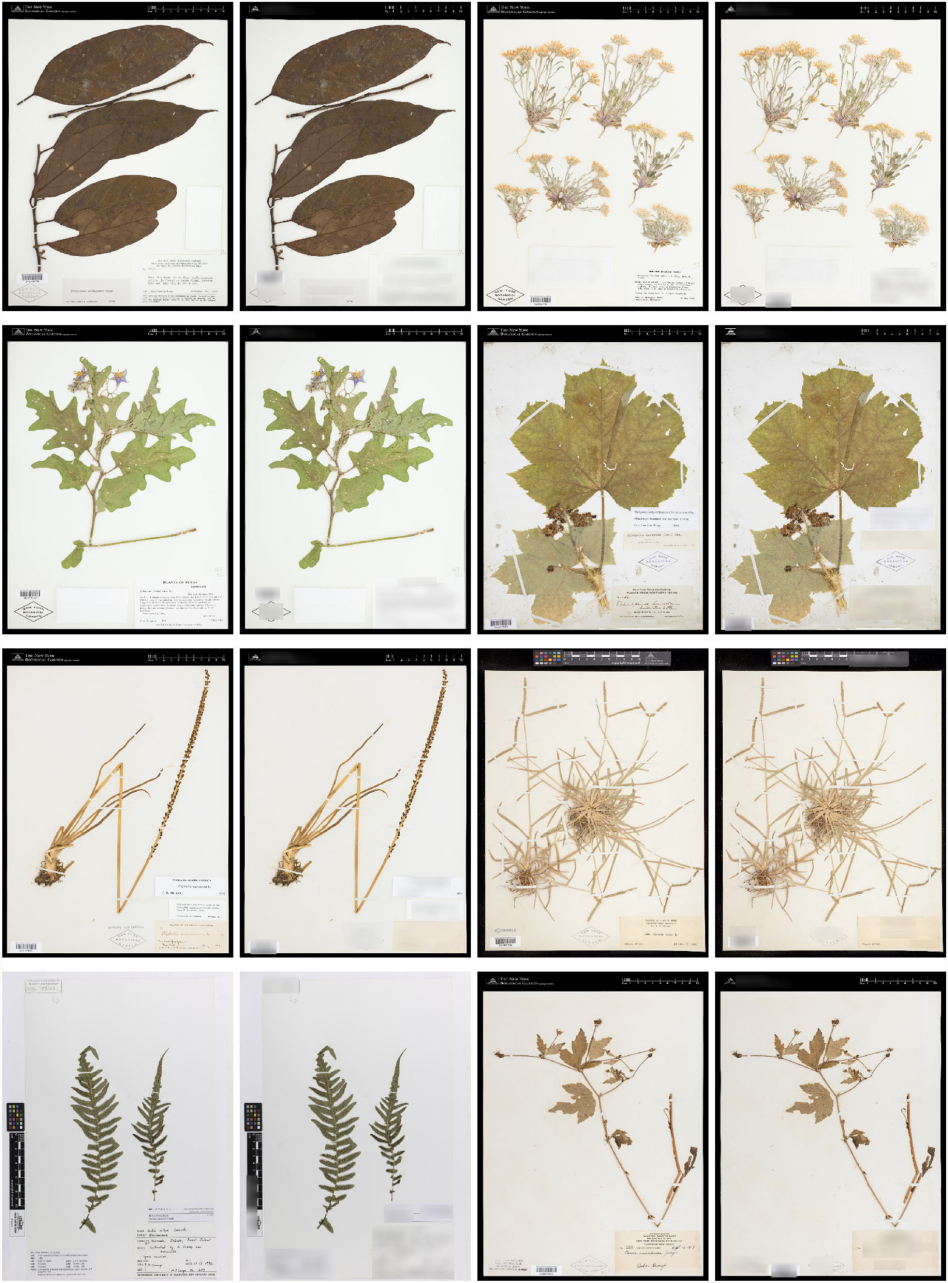
Example of images before **(left)** and after **(right)** preprocessing.

In this section, we give an overview of how the Herbarium 2021 Half–Earth dataset was preprocessed. Figure 6 presents some example herbarium sheet images before and after the preprocessing steps.

##### 2.1.2.1. Label Alignment

Herbarium specimens of the same taxon may have been labeled in various ways due to differences in the interpretation of taxon circumscriptions, nomenclature changes, and/or errors. For example, over time *Pilosella piloselloides* (Vill.) Soják (Asteraceae) has been known by at least 526 different names (Freiberg et al., 2020). To ameliorate this situation as much as possible, image labels are standardized to the Leipzig Catalogue of Vascular Plants (LCVP v1.0.2; Freiberg et al., 2020). Labels in the dataset have an LCVP status of either “accepted” or “unresolved”. The data exported from the institutional databases were first processed to find labels that exactly matched LCVP. For labels that did not precisely match, we then searched for long unambiguous partial matches to LCVP: the label was shortened by removing the rightmost word and then searched for a match that produced only one LCVP output taxon; if no match was found, this was repeated until the label contained only two words. Labels that still did not unambiguously match LCVP, were matched using tre-agrep (Wu and Manber, 1992) allowing an increasing amount of mismatch (10–30% of label length; all weights were set to 1). Matches returned by tre-agrep were manually reviewed (8,430 labels passed manual review). Images with labels that could not be coerced into matching LCVP were excluded from the dataset (*c*. 73 thousand images).

##### 2.1.2.2. Image Blurring

Herbarium specimens always have a hand–written or printed label on the sheet (usually lower right–hand corner), which includes information about the name of the taxon, the geographic location where it was collected, the date of collection, and the person or team of people who collected it. In addition, annotation labels are often added to the specimen to correct or update information on the original label—these are sequentially added in the empty space above the original label. Specimens often also have institutional labels or stamps indicating the herbarium in which the specimen is archived and a barcode label corresponding to an institutional database entry. Specimens may also include field tags with identification numbers attached directly to the plant. Images usually include color and measurement scales as well as institutional logos. All of these labels can of course, help identify the specimen, thus this information in the dataset was blurred in order to force models to learn about the plants themselves rather than the label text. A pretrained EAST text detection model (Zhou et al., 2017) was used to detect these labels. This model outputs bounding boxes around the detected text. The bounding boxes that overlapped by a sufficient margin were merged and those that were too small were filtered out. The resulting regions were then heavily blurred. First, a mean blur was applied, then a single Gaussian blur with added noise, and then a smooth alpha map to blend into the original (Figure 6). Finally, images where more than 25% of the image was blurred were excluded from the dataset, as those represent, in most cases, wrong predictions from EAST. The text detection model was deliberately tuned to have a high specificity, in order to avoid unnecessarily blurring plant parts. Even though, this means that there are images where part of the labels are missed by the blurring algorithm.

##### 2.1.2.3. Image Resizing

Herbarium sheets are digitized as very high–resolution images to preserve as much of the detail as possible. A common image size is around 6000 × 4000 pixels. This is very large even for networks that are designed to work with higher resolutions. All images in the dataset are resized to a dimension of 1,000 pixels (while preserving the aspect ratio), in order to make the overall size of the dataset more accessible.

##### 2.1.2.4. Dataset Split

Herbarium 2021 contains images from 64,500 taxa at the species–level or below with 2,257,759 in the training set and 243,020 in the test set. The data has been split to obtain an approximately even number of images across taxa in the test set by capping the maximum number of images per taxon at 10. For taxa that have few images a 80%/20% split for training/test is used—each category has a minimum of three images: at least one in the test set and two in the training set.

##### 2.1.2.5. Hierarchical Labels

In addition to the name of the taxon, labels for the family and order are provided. The herbarium sheet images provided in this dataset represent 64,500 different taxa, belonging to 451 families and 81 orders. This enables the development of methods that utilize hierarchical information. Ideally, mistakes between closely related taxa should not be treated equal to mistakes between very distant taxa. See Section 2.2 for an example of a loss function that leverages hierarchical labels.

### 2.2. Baselines

In order to have a reference value for the dataset performance, a standard ResNet-50 (He et al., 2016) was trained as a baseline method. A balanced sampling strategy was used to mitigate the impact of the imbalance on the classifier. The images were resized to 256 × 256 pixels and standard data augmentations were applied (small rotations, horizontal flips, color–jitter, and center–crop to 224 × 224 pixels). The model was initialized with weights pretrained on ImageNet (Deng et al., 2009). Finally, the model was trained using the standard cross–entropy loss, a batch size of 32, a stochastic gradient descent with a learning rate of 1 × 10^−3^ which is further reduced when a plateau was reached and a momentum factor of 0.9. The model was trained for a total of 10 epochs (with 70,555 batches per epoch).

To integrate hierarchical labels, the marginalization loss function proposed in Kumar and Zheng (2017) was adopted. The basic idea behind the marginalization loss is to simultaneously apply a classification loss at all the levels of the hierarchy. In order to compute the marginalization loss the label and the predicted distribution at each level of the hierarchy are needed: the label can simply be obtained by looking up the family and order; the predicted distribution for the family (or order) can be estimated from the sum of scores for all the taxa in each family (or order) in what resembles a marginalization procedure. Note that if the network predicts a distribution over the taxa, the marginalization over family and order also leads to a valid categorical distribution. A cross–entropy loss at the taxa level as well as the family and order level of hierarchy can be applied—this should ideally improve the regularization power of the network.

### 2.3. Evaluation Metrics

In order to evaluate the classification performance the main metric chosen for the Herbarium 2021 challenge was the F_1_ score, which is equal to:


(1) $$\begin{array}{l} {\text{F}}_{1}=2\frac{\text{Pre }\cdot \text{ Rec}}{\text{Pre }+\text{ Rec}}, \end{array}$$

where Pre denotes the precision and Rec the recall. This score is computed for every taxon separately and then averaged across all taxa to get the final score. Accuracy Acc and mean class accuracy Mca (also know as per–class accuracy) are also reported.

As an additional performance metric, the patristic distance between the expected and the predicted classes is also reported. Patristic distances were extracted from a dated genus–level phylogeny pruned to include only the taxa in the dataset (Jin and Qian, 2019). Within genera, distances among taxa were crudely interpolated by adding 10% of the distance between each genus and its sister genus.

## 3. Results

### 3.1. Competition Results

**Figure 7:**
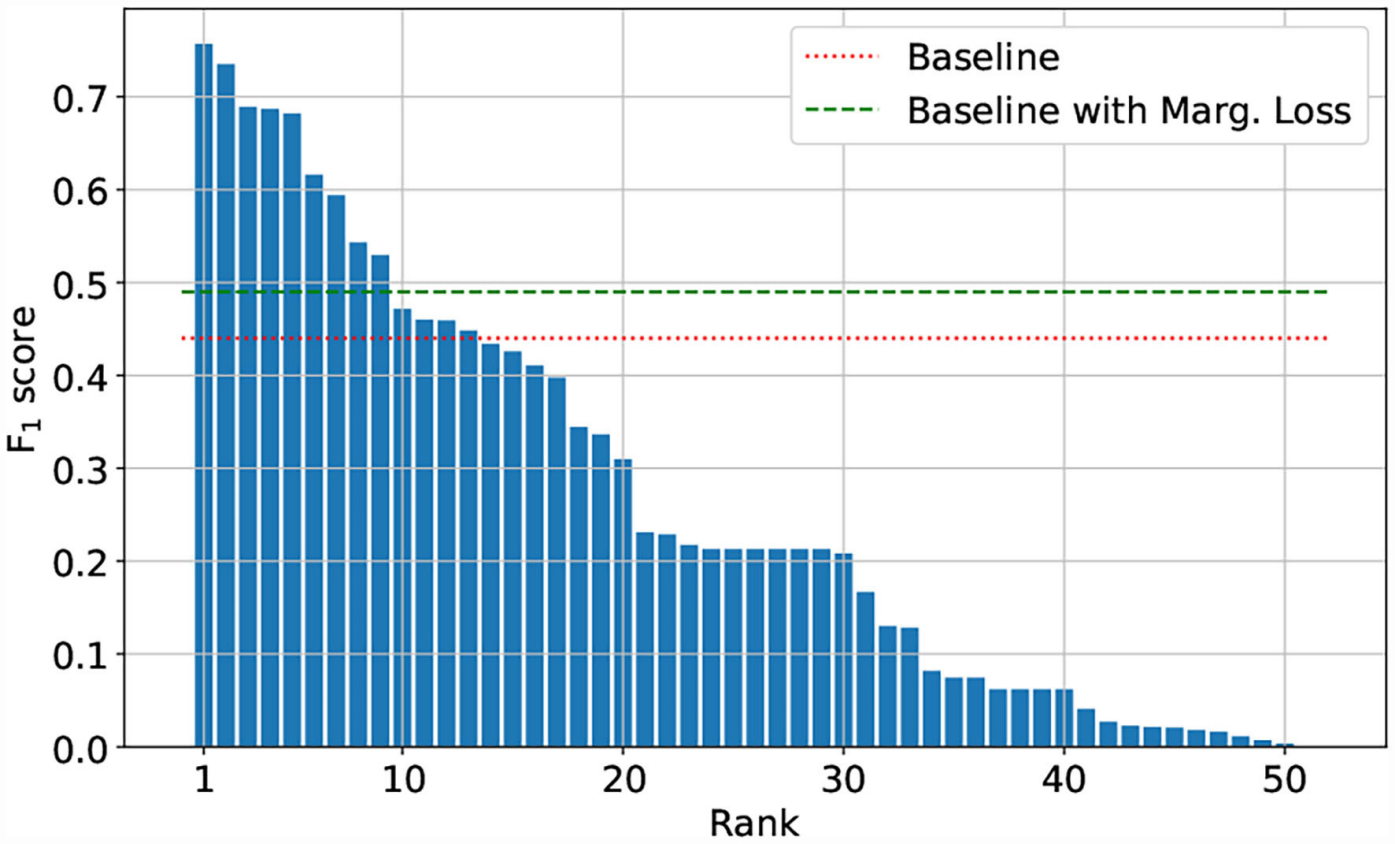
F_1_ scores of the 50 best performing teams.

The Herbarium 2021 Half–Earth challenge received 573 entries submitted by 108 competitors divided across 80 teams. As seen in Figure 7, there are large gaps in performance between the competitors. Focusing on the top–five teams of the competition: all had F_1_ performance above 0.680 on the test set. The teams are (in order of decreasing F_1_ scores): CIPP (0.757), HaeC (0.735), Brendan Rapazzo (0.689), Qidian213 (0.687), Undergrad & Botany Joe (0.682; Table 2). All of the top–five approaches used relatively high resolution images (352 × 352 pixels or higher). The top–three solutions were ensembles of models, with the top–two teams combining the predictions from different models and the third place team combining predictions made by the same model at the different stages of the training process.

**Table 2:** Summary of the top competitors' solutions and performance.

**Team ranking**	**1st**	**2nd**	**3rd**	**4th**	**5th**
Team name (Organization)	CIPP (Alibaba Group)	HaeC (Postech)	Brendan Rapazzo (Cornell University)	Qidian213	Undergrad & Botany Joe (The University of Tennessee)
Team members	Baoming Yan, Bo Gao, Xiao Liu, Lin Wang, and Chao Ban		Brendan Rapazzo	—	Dax Ledesma and Joey Shaw
Model architecture	ResNest101, ResNeXt101-IBN-a, ResNeXt101	TResNet-M, TResNet-M-21k, TResNet-L, GENet-L, ECA-NFNet-L0	SE-ResNeXt101	—	SE-ResNeXt50
Feature extractor parameters (M)	48.3+89+89 = 226.3	29.4+29.4+54.7+31+24 = 169.5	95	—	28
Input image resolution	256 × 256, 256 × 256, 352 × 352	448 × 448	448 × 448	—	448 × 448
Loss function	Triplet, AM-softmax, LDAM	SoftTriple, Cross-entropy, BM-Softmax.	Cross-entropy	—	Cross-entropy
F_1_	0.757	0.735	0.689	0.687	0.682
Acc	0.845	0.837	0.793	0.799	0.786
Mca	0.787	0.761	0.706	0.725	0.693

*Note that the fourth place team did not respond to the post–competition survey. We define as a feature extractor the part of the model that extracts the feature maps on which the classification is based. The feature extractor parameters are taken from the publications associated with the respective model architectures*.

Collectively, the top–five teams used seven different base neural network architectures:

- **ResNeXt** The ResNeXt architecture introduced by Xie et al. (2017) is a popular network architecture that extends ResNet (He et al., 2016). It leverages the split–transform–merge (proposed in Inception; Szegedy et al., 2015) to split the input into multiple blocks and then merge those blocks after convolution.
- **ResNeXt-IBN-a** Pan et al. (2018) proposed IBN-Net as an extension to any existing network—in this case the ResNeXt architecture. IBN stands for Instance and Batch Normalization—the main modifications used in IBN-Net to achieve domain/appearance invariance. This modificaiton is a simple way to increase both modeling and generalization capacity without increasing computational burden.
- **SE-ResNeXt** The SE network introduced by Hu et al. (2018) focuses on channel relationships instead of the spatial component of convolutional blocks. This is done by using the “Squeeze–and–Excitation” (SE) block, that adaptively recalibrates channel–wise feature responses by explicitly modeling interdependencies among channels. In this case the standard convolutional blocks in the ResNeXt architecture are replaced by these new SE blocks.
- **ResNeSt** The ResNeSt architecture proposed by Zhang et al. (2020) is a variant of the ResNet model which instead stacks Split–Attention blocks which are effectively channel–wise attention on different network branches.
- **TResNet** The TResNet architecture proposed by Ridnik et al. (2020) is designed to be highly efficient in training time and inference time while achieving a better performance than a comparable ResNet.
- **ECA-NFNet-L0** The ECA-NFNet is a variant of the Normalization–Free neural Network (NFNet; Brock et al., 2021) with Efficient Channel Attention (ECA) layers (Wang et al., 2020) instead of SE blocks, which results in one third of the number of parameters of the original NFNet.
- **GENet** The GENet proposed by Lin et al. (2020) is designed to be efficient when trained on a GPU. In fact, it achieves a similar performance, but is up to 6.4 times faster than EfficientNet (Tan and Le, 2019).

Interestingly, the top–two teams leveraged recently proposed deep metric learning losses in addition to the standard cross–entropy loss used for classification. The goal of deep metric learning is to learn an embedding where the features extracted from examples of the same class (in this case, the same taxon) are closer than the ones extracted from examples of different classes. The issue with standard cross–entropy loss preceded by a softmax is that it learns separable features that are not discriminative enough—this problem is exacerbated in the Herbarium 2021 dataset where the training set is extremely long–tailed and performance is measured on a relatively well-balanced test set. One way to produce a deep metric learning embedding is to cast it as an optimization problem with triplet constraints, which correspond to the Triplet loss: learning is performed on a set of three images, the anchor (the baseline image), the positive image (another image belonging to the same class as the anchor), and the negative image (an image belonging to a different class). The goal is then to have features which correspond to the anchor and the positive image (or images) close in the embedding space while the anchor and the negative image (or images) are far in the embedding space. However, this procedure is time consuming and it is very sensitive to the selection of anchor, positive, and negative images. As a result there has been a number of loss functions proposed as extensions of the standard cross–entropy loss, that achieve the objective of the distance metric learning paradigm without having to compare multiple image samples in embedding space: Additive Margin Softmax loss (AM–softmax; Wang et al., 2018), Balanced Meta–Softmax loss (BM–softmax; Ren et al., 2020), and SoftTriple loss (Qian et al., 2019) are examples. Finally the Label–Distribution–Aware Margin Loss (LDAM; Cao et al., 2019) is designed to replace the cross–entropy loss—it is designed specifically for the case in which the training dataset is heavily imbalanced while the testing criterion requires good generalization on less frequent classes.

Regarding the losses, unfortunately none of the teams leveraged the provided hierarchical labels. In Table 3, we highlight the potential increase in performance that could be achieved by using them. In fact, there is clearly a substantial improvement when comparing the performance of the baseline model trained with a standard cross–entropy loss to the performance achieved when training the same model with the marginalization loss (Section 2.2). The marginalization loss is trivial to extend to any of the loss functions used by the competitors other than cross–entropy loss (Table 2).

**Table 3:** Ablation study for marginalization loss utilizing hierarchical label information.

**Model**	**F_**1**_**	**Acc**	**Mca**
Baseline	0.442	0.543	0.485
Baseline with marginalization loss	0.494	0.599	0.534

### 3.2. Performance on Difficult Examples

The top–five competition models accurately predicted the correct taxa for the examples presented in Section 2.1.1: *Abarema brahcystahya* (Figure 3), used to illustrate different reproductive stages, had an average top-1 accuracy of 0.914 (test images *n* = 10; training images *n* = 33); *Arbutus xalapensis*, used to illustrate variation in specimen preparation (Figure 4) had an average top-1 accuracy of 0.94 (test images *n* = 7; training images *n* = 297); and the *Alyssum* species, used to illustrate similar morphology among closely related taxa (Figure 5), had an average top-1 accuracy of 0.926 (test images *n* = 38; average training images per species *n* = 40.22, range = 2–154).

### 3.3. Patristic Classification Error

**Figure 8:**
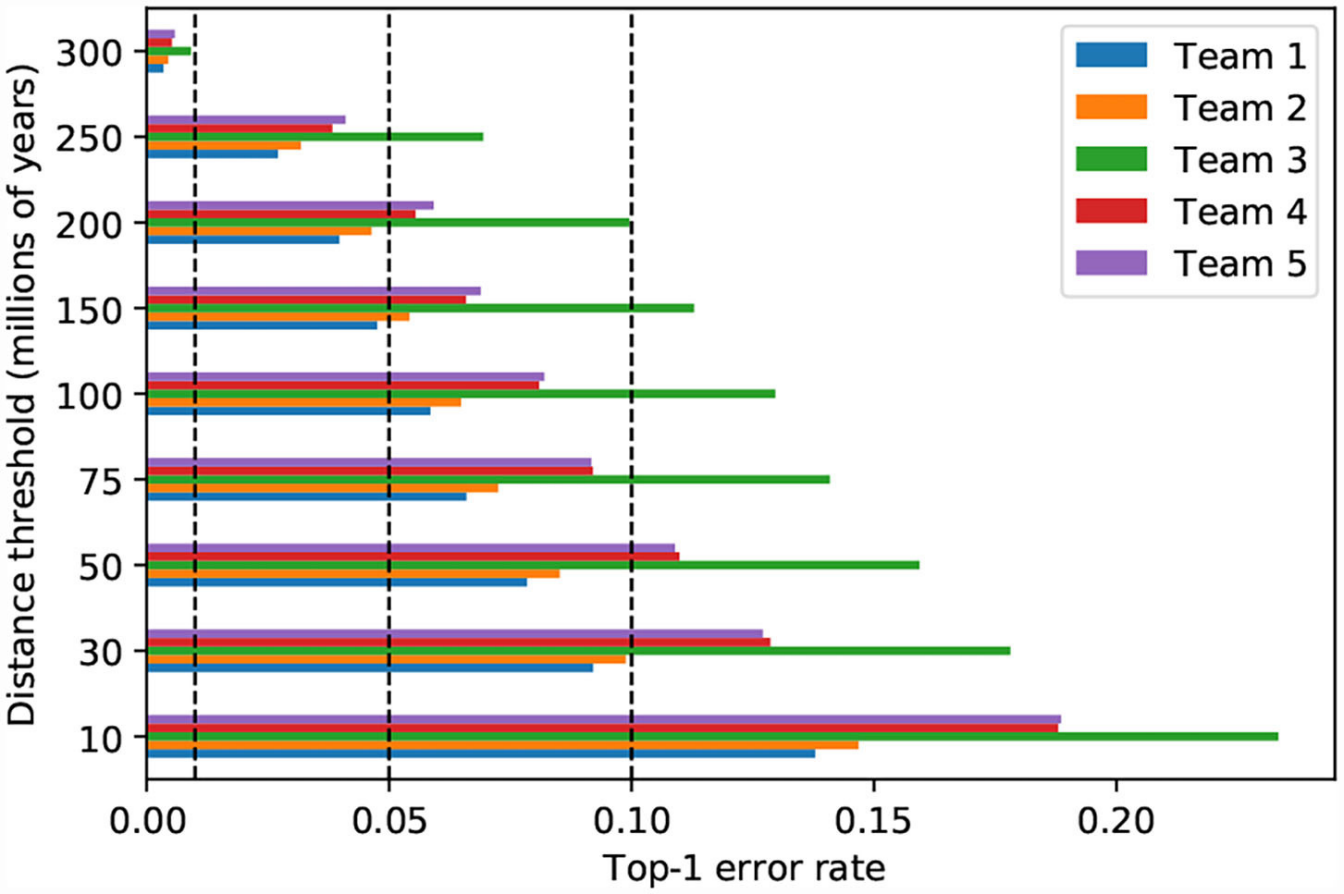
Model performance measured by different phylogenetic proximity thresholds. Top-1 error is calculated by counting all predictions that are within the patristic distance threshold as successes. The vertical dashed lines represent top-1 error at 0.01, 0.05, and 0.10, respectively.

The magnitude of classification error can be measured by the patristic distance between the expected and predicted taxa. When the predictions of the top–five models are incorrect, the wrongly predicted taxon is usually one that is phylogenetically close to the expected taxon (i.e., low patristic distance between predicted and expected taxa). For instance, if all model predictions within a maximum patristic distance of 10 million years (My) from the expected taxon are considered correct, then all five top models display similar accuracy (0.77–0.86; Figure 8). On the other hand, when the threshold is 30 My, which is close to the median patristic distance between sister genera (31.628 My), the error rate is less than 10% for the top–two models (Figure 8). Thus, the models are generally correct at the genus–level and more than half of the original error is due to incorrect classification of taxa within genera.

When evaluated in the light of patristic classification error, the third place model does not appear to behave like the other top–five models (Figure 8): perhaps the features it extracts are less correlated with phylogeny than the features extracted by the other top models. Given that the fifth place model uses the same SE-ResNeXt base architecture and cross–entropy loss function, the deviant performance could, perhaps, be attributed to training parameters.

**Figure 9:**
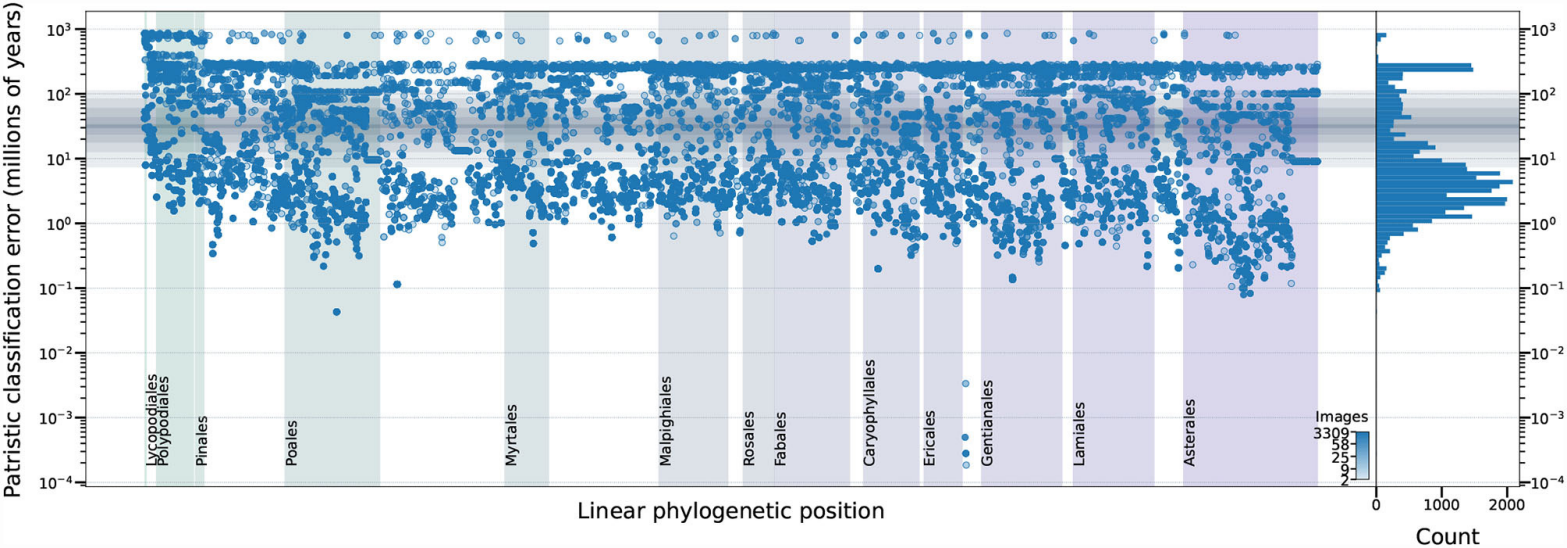
The relationship among dataset properties and incorrect model predictions for the top performing model. The phylogenetic relationship among the expected taxa (x–axis) is represented by the right ladderized phylogenetic tree for all genera in the dataset (Jin and Qian, 2019). The y–axis indicates the identification error—expressed as patristic distance between the expected and predicted classifications. The number of training images for each expected taxon is indicated by marker color and visualized as a histogram in the right panel. The median patristic distance between sister genera is represented by a solid horizontal gray line with gray boxes indicating the 10–90, 20–80, 30–70, and 40–60 decile ranges. The top ten angiosperm, top gymnosperm, top fern, and top lycophyte orders, as measured by the number of training images, are labeled. Results for 628 of 243,020 (0.26%) test images are not displayed because those taxa could not be located in the reference phylogenetic tree (Jin and Qian, 2019).

Examination of the erroneous predictions made by the top performing model do not indicate any phylogenetic clustering of errors—demonstrating that the top model performs equally well (or equally poorly) on all types of plants in the dataset (Figure 9). If a botanist was to be provided with the low–resolution input images used by the top model, they would be unlikely to perform as uniformly as the model: taxa in some orders are almost exclusively differentiated by features occupying only a fraction of a pixel at that resolution (e.g., Poales) while taxa in other orders are more easily differentiated at that resolution (e.g., Rosales).

Prediction errors less than the median patristic distance between sister genera (31.628 My; solid horizontal gray line in Figure 9), are the sorts of errors that botanists commonly make (i.e., misidentifying taxa within genera). Some of these model errors may be the result of uncaught labeling errors in our dataset. Prediction errors above the 90*th* decile of the patristic distance between sister genera (112.160 My; outer gray box in Figure 9) are errors that botanists rarely make and, thus, are unlikely to be attributable to incorrect dataset labeling.

### 3.4. Factors Contributing to Prediction Error

**Figure 10:**
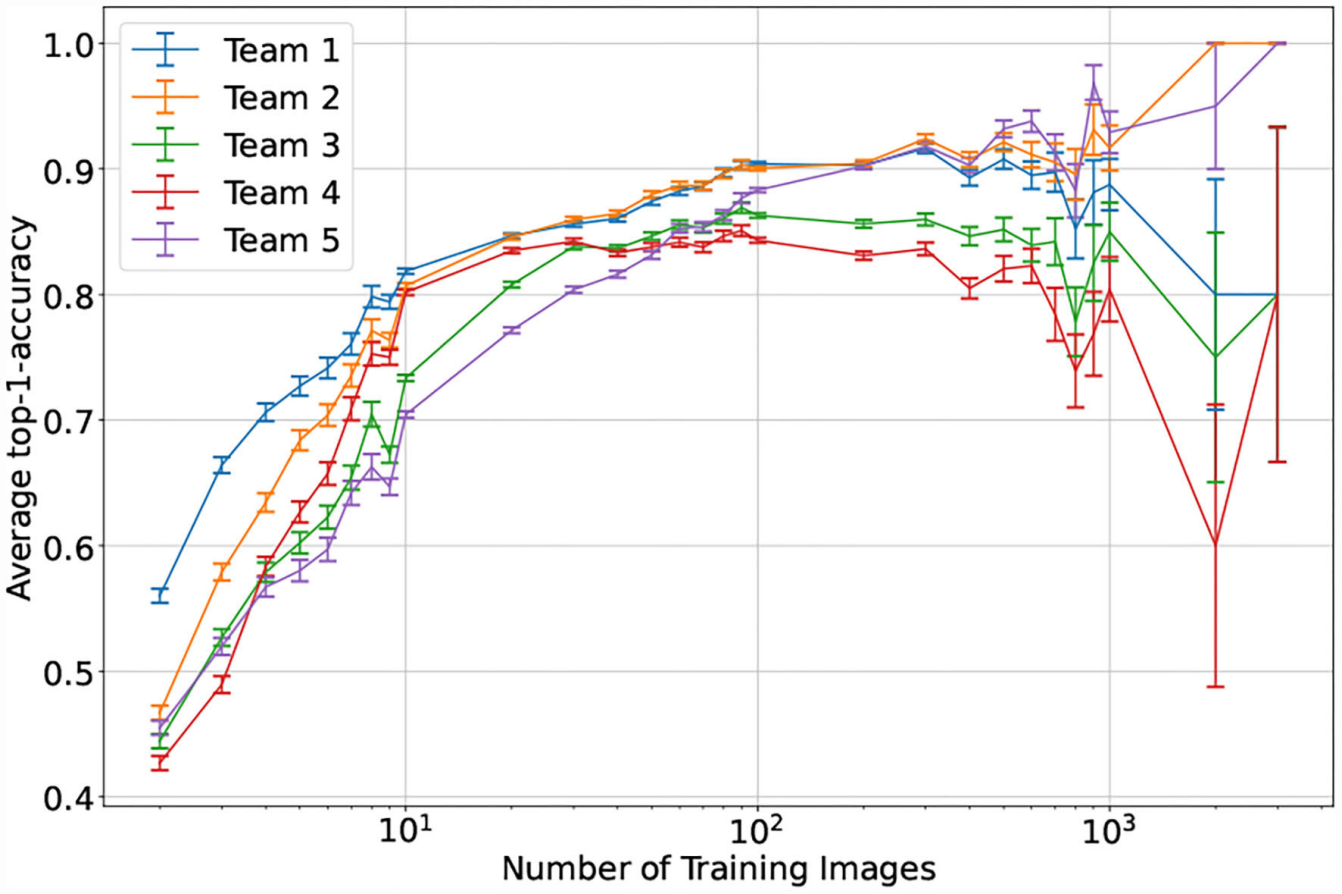
Mean performance of the top–five models by number of training images. Taxa are aggregated based on their corresponding number of training images. The test time performance is then visualized as the mean of the top-1-accuracy for all taxa in a specific bin, the error bars correspond to the average standard error for each bin.

For the top performing model, the number of expected taxon training images appears to be associated with prediction failure, but the relationship is not absolute: there are cases, particularly common in the Polypodiales, in which the number of training images is high (dark blue circles in Figure 9) and the patristic classification error is high. The relationship between model accuracy and number of training images is more straightforward (Figure 10): the top performing model shows poor accuracy for taxa with only two training images (accuracy = 56.0%, *n* = 7,745), while the accuracy substantially increases with more training images (e.g., accuracy = 79.8% for taxa with eight training images, *n* = 2,147). The top-1 accuracy of the second and fifth place models is less than 50% (42.7–46.7%, *n* = 7,745) with two training images, while a similar boost in accuracy with more training data is observed (66.3–77.1% with eight training images, *n* = 2,147). Model accuracy increases with the number of training images to different degrees across the top–five models. The top–two model shows a consistent boost in its performance as training images increases (*n* = 2–3309), whereas other models display inconsistent performance boosts after *n* = 100 training images—the top–four model shows a consistent decrease in performance after *n* = 100 training images.

Another factor affecting model predication accuracy is specimen quality: we examined the 144 specimen images in the test dataset that produced egregiously incorrect (i.e., patristic classification error greater than 600 My) predictions from the top performing model, and compared them to an equally sized randomly–sampled set of images with correct predictions. About 9.1% of the egregiously misclassified images were not good plant specimens: 0.7% were photographs of living plants, 0.7% were botanical illustrations, 3.5% lack plant materials entirely, and 4.2% were closed packets that obscured all plant materials from view. None of the correctly predicted specimen images had the above issues. Among the egregiously misclassified specimen images with visible plant materials (90.9%), nearly half (46.6%) consist entirely of plant fragments (e.g., single organs like fruits, buds, leaves, inflorescence, etc.) and a quarter (25.7%) are diminutive plant taxa—that remain small at full maturity—and therefor cover only a tiny fraction of specimen image. In contrast, 3.6% of correctly predicted specimen images consist entirely of plant fragments and none were diminutive plants.

## 4. Discussion

### 4.1. Competition Results

The Herbarium 2021 Half–Earth challenge is the richest plant dataset in the world for fine–grained visual categorization, but it pushes the limits of contemporary machine learning—automated herbarium specimen image classification is a challenge that involves differentiating among plant taxa with subtle differences in color, texture, and shape. When compared to other fine–grained image datasets such as ImageNet (distinct classes easily classified by the general public; Deng et al., 2009) or iNaturalist (distinct classes that are easier to classify due to their spread across different kingdoms of life; Horn et al., 2021), the difficulty of classifying herbarium specimen images is apparent. The high number (64,500) and imbalanced distribution (imbalance factor = 1,654.5) of classes in the Herbarium 2021 dataset, makes this task especially challenging given the numerous classes with few images—nearly half (49.1%) of the taxa have less than 10 training images. Despite these challenges, the deep learning models submitted to the competition demonstrated performance far beyond our expectations: macro F_1_-score = 0.76 and top-1-error = 15.5%.

Most recently ViT-G/14 (Dosovitskiy et al., 2021) achieved a top-1-error of 9.55% on ImageNet—the most widely used image classification dataset. Considering that our dataset is much more unevenly distributed and has 60 times more classes than ImageNet,the top-1-error of 15.5% for the Herbarium 2021 Half–Earth challenge is quite remarkable (Table 2). For taxa with more than 50 training images (*n* = 10,355 taxa), the top-1-error (10.4%) of the top performance model is comparable to the state-of-the-art top-1-error of ImageNet (9.55%)—even with 10 times more classes and 7.8 times fewer parameters than ViT-G/14 (230 M vs. 1,800 M). The iNaturalist 2021 (Horn et al., 2021) fine–grained visual categorization dataset is similar to Herbarium 2021 in many ways, but it includes only ten thousand taxa with a more balanced training data distribution (>100 training images per taxon) and incorporates image geo–locations. In contrast, Herbarium 2021 does not include collection locations. In the Kaggle competition, the best model for iNaturalist 2021 had a top-1-error rate of 4.4%. If the Herbarium 2021 dataset had a more balanced distribution of training images, we see it having the potential to become another rich source of fine–grained visual categorization tests.

Finally we would like to point out that the competition was particularly difficult for competitors who did not have the computational resources to train large models on this amount of data—training a large model on this dataset is quite time consuming: for example training the baseline model took around 120h on an NVIDIA Titan X GPU. As can be seen in Table 2, the top-5 competitors' performance seems to be correlated with the number of parameters of the feature extractor.

### 4.2. Future Directions

There a multiple future directions that can be explored within the scope of fine–grained herbarium classification:

- Automated analysis of digitized natural history collections may help reduce the bottlenecks in identifying new species held in collections: herbaria are thought to already house specimens of half of the plant species that have not yet been formally described in the scientific literature (Bebber et al., 2010). There is an incredible backlog in specimen identification and curation in herbaria and many lack staff and taxonomic expertise to readily identify all of their specimens. With this urgent need in mind, we believe that there is an opportunity to facilitate the work of botanical experts to enable them to focus on the most critical tasks that cannot be automated. One useful approach may be to build a dataset that includes unlabeled data so that competitors could explore approaches related to semi–supervised learning or active learning rather than limiting competitions to straightforward supervised learning tasks. Furthermore, systems to accurately estimate well-calibrated uncertainties linked to the taxon prediction task would be extremely useful to make sure that we prioritize specimens most needing attention from expert botanists.
- It may be possible to leverage the digitized data stored in the herbaria to classify pictures of living plants. Overcoming the distribution shift between training on herbarium sheet images and testing on images of live plants is non-trivial, nevertheless recent advancements in generative models and domain adaptation can be effectively applied to such a scenario.
- Future Kaggle challenges should encourage engagement between different research communities, such as computer vision scientists and botanists. Computer vision scientists often adopt an approach aimed at maximizing algorithm performance in terms of the evaluation metrics, but they may be unaware of domain specific knowledge, such as the patristic distance, that can be used to both improve model interpretability and performance. On the other hand, botanists may not be aware of the latest advances in computer science that may boost model performance.
- Although large datasets increase the difficulty of the competition and push the boundaries of automatic taxon recognition, they exclude participants without access to a large computational resources for training machine learning models. As a result, future Kaggle challenges could be designed so that they can be split in multiple parts with at least some of the parts computationally accessible to all (e.g., a dataset of selected families or orders, or a dataset with a cap on the maximum number of images per taxon).

## 5. Conclusion

We have created the Herbarium 2021 Half–Earth dataset to enable the development of better automatic taxon recognition models. The development of models to automatically identify specimens will reduce the species identification bottleneck and has the potential to improve both the quality and accelerate the pace of biodiversity research.

In the future, we would like to expand the dataset to include specimens collected world–wide. There are more than 35 million digitized specimens in electronic databases representing more than 80% of the known vascular plant diversity.

## Data Availability Statement

The datasets presented in this study can be found in online repositories. The names of the repository/repositories and accession number(s) can be found in the article/supplementary material.

## Author Contributions

RL, DL, JP, KW and SD'A wrote the manuscript in consultation with JDW, SB, FM, and BA. RL and DL prepared the dataset and the competition in consultation with JDW, SB, FM, and BA. KW, JJW, MT, RP, TG, GB, GG, AF, DR, and YB selected and provided the data from their respective institutions. SB and BA conceived the original idea. All authors contributed to the article and approved the submitted version.

## Funding

This work was partially funded by National Science Foundation (USA) grant DEB 2054684.

## Conflict of Interest

The authors declare that the research was conducted in the absence of any commercial or financial relationships that could be construed as a potential conflict of interest. The handling editor declared a shared research group [FGVC virtual lab] with one of the authors [SB] at time of review.

## Publisher's Note

All claims expressed in this article are solely those of the authors and do not necessarily represent those of their affiliated organizations, or those of the publisher, the editors and the reviewers. Any product that may be evaluated in this article, or claim that may be made by its manufacturer, is not guaranteed or endorsed by the publisher.

## Footnotes

^1^ https://www.kaggle.com

^2^ https://github.com/visipedia/herbarium_comp

^3^Images of specimens from American Samoa, Anguilla, Antigua and Barbuda, Argentina, Aruba, Australia, Bahamas, Barbados, Belize, Bermuda, Bolivia, Brazil, Canada, Cayman Islands, Chile, Colombia, Cook Islands, Costa Rica, Cuba, Curaçao, Dominica, Dominican Republic, Ecuador, El Salvador, Falkland Islands, Fiji, French Guiana, French Polynesia, Greenland, Grenada, Guadeloupe, Guatemala, Guyana, Haiti, Honduras, Indonesia (island of New Guinea only), Jamaica, Kiribati, Marshall Islands, Martinique, Mexico, Micronesia, Montserrat, Nauru, New Caledonia, New Zealand, Nicaragua, Niue, Norfolk Island, Northern Mariana Islands, Palau, Panama, Papua New Guinea, Paraguay, Peru, Philippines, Pitcairn, Puerto Rico, Saint Barthélemy, Saint Kitts and Nevis, Saint Lucia, Saint Martin, Saint Pierre and Miquelon, Saint Vincent and the Grenadines, Samoa, Solomon Islands, Suriname, Tokelau, Tonga, Trinidad and Tobago, Turks and Caicos Islands, Tuvalu, United States Minor Outlying Islands, United States of America, Uruguay, Vanuatu, Venezuela, Virgin Islands (both British and US), and Wallis and Futuna were included in the dataset.
